# Widely tunable black phosphorus mid-infrared photodetector

**DOI:** 10.1038/s41467-017-01978-3

**Published:** 2017-11-22

**Authors:** Xiaolong Chen, Xiaobo Lu, Bingchen Deng, Ofer Sinai, Yuchuan Shao, Cheng Li, Shaofan Yuan, Vy Tran, Kenji Watanabe, Takashi Taniguchi, Doron Naveh, Li Yang, Fengnian Xia

**Affiliations:** 1 Department of Electrical Engineering, Yale University, New Haven, CT 06511 USA; 2 Department of Physics and Institute of Materials Science and Engineering, Washington University, St. Louis, MO 63130 USA; 3 Faculty of Engineering and Bar-Ilan Institute for Nanotechnology and Advanced Materials, Bar-Ilan University, 52900 Ramat-Gan, Israel; 4 Advanced Materials Laboratory, National Institute for Materials Science, 1-1 Namiki, Tsukuba, 305-0044 Japan

## Abstract

Lately rediscovered orthorhombic black phosphorus (BP) exhibits promising properties for near- and mid-infrared optoelectronics. Although recent electrical measurements indicate that a vertical electric field can effectively reduce its transport bandgap, the impact of the electric field on light-matter interaction remains unclear. Here we show that a vertical electric field can dynamically extend the photoresponse in a 5 nm-thick BP photodetector from 3.7 to beyond 7.7 μm, leveraging the Stark effect. We further demonstrate that such a widely tunable BP photodetector exhibits a peak extrinsic photo-responsivity of 518, 30, and 2.2 mA W^−1^ at 3.4, 5, and 7.7 μm, respectively, at 77 K. Furthermore, the extracted photo-carrier lifetime indicates a potential operational speed of 1.3 GHz. Our work not only demonstrates the potential of BP as an alternative mid-infrared material with broad optical tunability but also may enable the compact, integrated on-chip high-speed mid-infrared photodetectors, modulators, and spectrometers.

## Abstract (web summary)

The bandgap of ultrathin black phosphorus can be tuned by a vertical electric field. Here, the authors leverage such electric field to extend the photoresponse of a black phosphorus photodetector to 7.7 μm, opening the doors to various mid-infrared applications.

## Introduction

Black phosphorus^1–8^ (BP) recently has gained significant attention from the optoelectronic community, due to its moderate bandgap in mid-infrared (mid-IR)^3^, its high carrier mobility^9–11^, and its intrinsic layered nature, which allows for the on-chip monolithic integration with electronics and various optical waveguides^12–14^. Previously, a number of BP photodetectors^12–20^ have been demonstrated. However, for BP photodetectors relying on efficient interband transitions, the cutoff wavelength is around 3.7 μm, which is determined by its bulk bandgap of around 0.33 electron volt^3^. Extending its operation into longer wavelength range can significantly improve its functionalities. Furthermore, if the light-BP interaction leveraging interband transitions can be dynamically tuned within a wide spectral range in mid-IR, optical devices beyond photodetectors, such as on-chip high-speed mid-IR optical modulators and spectrometers, may be built based on this concept of dynamic response tuning. Such high-speed mid-IR optical modulators and spectrometers can play an important role in many applications, including free-space communications, sensing, and surveillance^14^.

Alloying arsenic with BP can form a new type of narrow gap semiconductor, black arsenic phosphorus (b-AsP)^21^. B-AsP has a bandgap even smaller than that of BP and b-AsP photodetectors show a decent photoresponse at mid-IR wavelength up to 8.2 μm^22^. On the other hand, recent transport studies in the BP thin film show that a vertical electric field can effectively reduce its transport bandgap by shifting its band edge energies^23–25^. In fact, such strong tuning of transport bandgap has also been observed in few-layer transition metal dichalcogenides^26^. However, light-matter interaction and carrier transport can respond to the external stimuli very differently in layered materials and it remains unclear how the external electric field can modify the light-BP interaction. In fact, in bilayer molybdenum disulfide (MoS_2_), the vertical electric field has been shown to significantly reduce its transport bandgap and the bandgap tuning is almost linearly dependent on the vertical electric field^26^. However, the light-matter interaction in a biased bilayer MoS_2_ is dominated by direct optical transitions within the same layer, which are almost field-independent^26^. On the contrary, at the direct bandgap (K point) in a strongly biased bilayer MoS_2_, the conduction and valence states are localized in different layers, respectively, leading to minimum optical transition dipole and weak light-matter interaction at the direct bandgap energy^26^.

In this work, we demonstrate widely tunable mid-IR photodetectors operating up to 7.7 μm based on a hexagonal boron nitride (hBN)/BP/hBN-sandwiched heterostructure. Here the hBN dielectric not only guarantees an ultra-clean interface for efficient photo-carrier collection but also prevents BP from oxidation. The high device quality allows for the observation of the strongest intrinsic photoconductive response near the charge-neutrality point in BP. The widely tunable photoresponse combined with theoretical calculations show the promising future of BP thin film as an alternative and high-quality material for mid-IR photonics especially for integrated photonic systems.

## Results

### Device fabrication and basic characterization

**Fig. 1:**
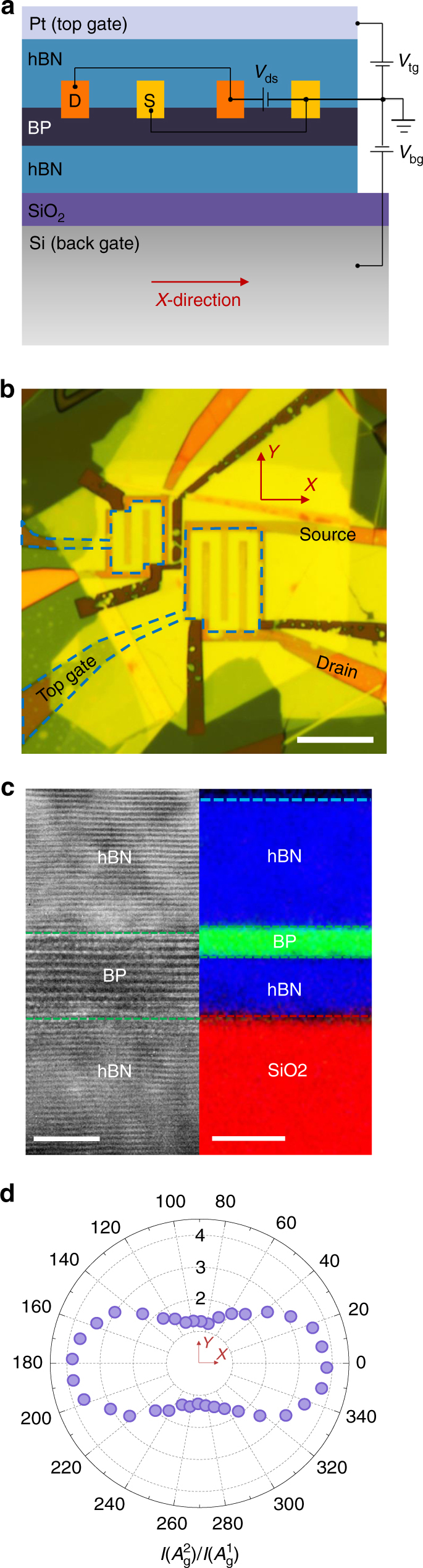
Structure of the tunable BP mid-IR photodetector based on a dual-gate transistor configuration. **a**, **b** Schematic (**a**) and optical (**b**) images of the dual-gate hBN/BP/hBN FET. Left device in **b** is used for the electrical and optoelectronic measurements. Scale bar: 10 μm. **c** The cross-section HR-TEM image of the hBN/BP/hBN stack. The thickness of BP is around 5 nm (10 layers). Right panel is the element analysis image for nitrogen (blue), phosphorus (green), and oxygen (red). Scale bars: 4/10 nm (left/right). **d** The ratio of Raman *A*_g_^2^ and *A*_g_^1^ peak intensity as a function of the excitation laser polarization. The ratio peaks when the excitation laser polarization is aligned along the *X*-direction (armchair) of BP

The schematic and optical images of the BP photodetector based on a dual-gate transistor structure are shown in Fig. 1a, b, respectively. Here, BP films are sandwiched by hBN flakes and transferred onto a silicon substrate covered with a 90 nm-thick silicon dioxide using the polymer-free dry transfer method^27^. All exfoliation and transfer processes are performed in an argon-filled glovebox with oxygen and moisture concentrations below 0.1 part per million (ppm) to prevent BP from oxidation. As shown in Fig. 1c, the cross-sectional high-resolution transmission electron microscopy (HR-TEM) image demonstrates the clean and oxidation-free BP/hBN interfaces. This is further evidenced by the elemental mapping (see Methods) for nitrogen (blue), phosphorus (green), and oxygen (red), as shown in the right panel of Fig. 1c. No oxygen signal is observed within the hBN/BP/hBN heterostructure. The thickness of BP, top and bottom hBN layers are also accurately determined from the cross-sectional HR-TEM. In this device, the BP thin film consists of 10 layers and the top and bottom hBN are 23 and 9 nm-thick, respectively. The top hBN dielectric consists of two hBN layers transferred separately. The first hBN layer is used to sandwich the BP film. The second hBN layer is transferred after the deposition of the interdigitated chromium/gold (3/27 nm) fingers for photocurrent collection, and it covers the channel and the electrodes. As a result, the second top hBN layer prevents the electrical shorting between the gate electrode and the interdigitated fingers for photocurrent collections. These interdigitated electrodes are designed along the *Y*-direction (zigzag) of BP thin flakes such that the carrier collection is along the *X*-direction (armchair), in order to achieve the highest carrier mobility and photo-responsivity. The BP crystalline direction was identified by the polarization-resolved Raman spectroscopy, in which the intensity ratio of peaks *A*_g_^2^ and *A*_g_^1^ reaches a maximum value of 3.8 when the polarization of excitation laser is aligned along the *X*-direction as shown in Fig. 1d ^28^. Detailed Raman analysis is presented in Supplementary Fig. 1 and Supplementary Note 1. Platinum thin film (~7 nm) is used as the partially transparent top gate with a mid-IR transmission efficiency of around 60% (Supplementary Fig. 2) and silicon is used as the back gate. The detailed device fabrication process is discussed in the Methods section and Supplementary Fig. 3.

### Transport measurement in a 10-layer BP device

**Fig. 2:**
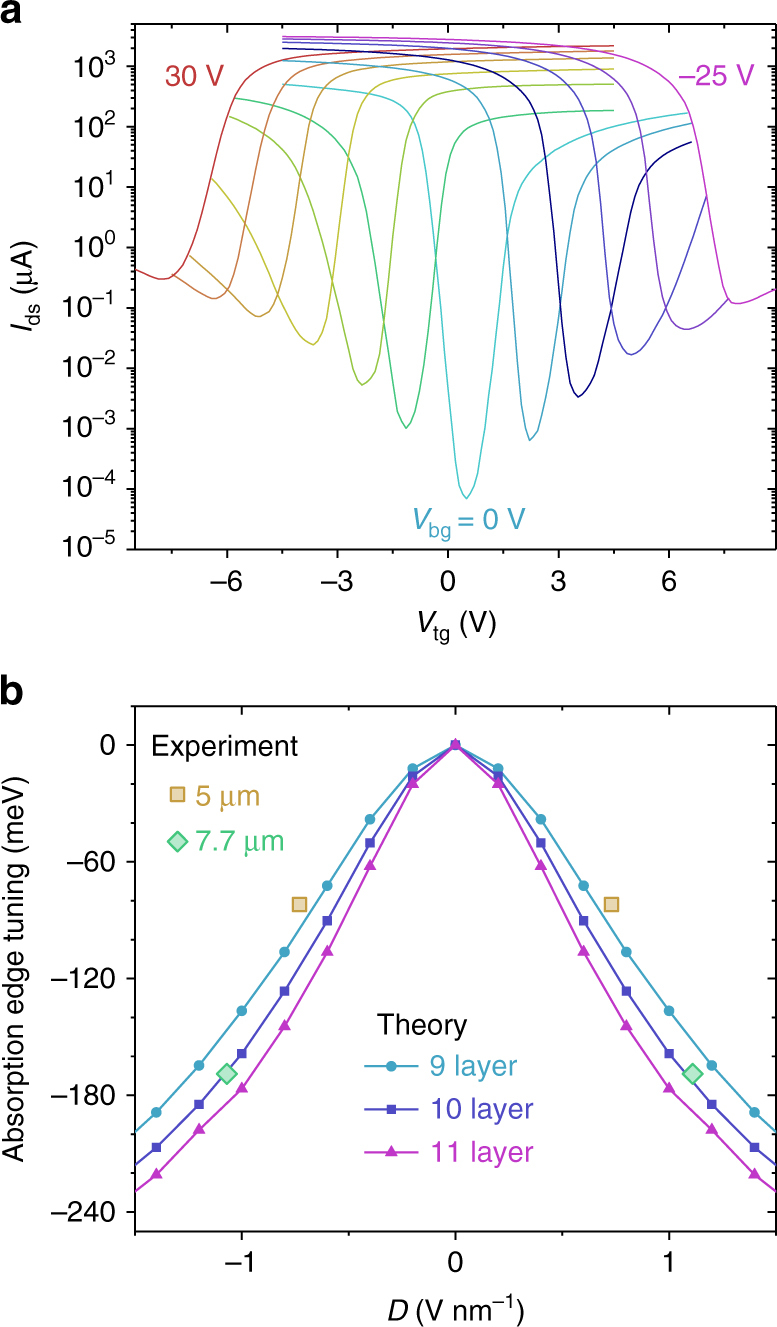
Electrical characterization and modeling of the 10-layer BP under vertical electric field. **a** Source-drain current of the dual-gate BP transistor as a function of top gate bias at different static back gate biases. The source-drain bias is 100 mV. **b** Modulation of the optical absorption edge in 10-layer BP under bias. Solid lines show the theoretical calculated optical absorption edge tuning as a function of displacement field in 9-, 10-, and 11-layer BP films. Experimental extracted results at 5 and 7.7 μm wavelength are plotted as scatters

We first performed two-terminal transport measurements as a function of top gate voltage *V*_tg_ in a 10-layer BP device (Fig. 2a) at different static back gate bias *V*_bg_ ranging from −25 to 30 V at 77 K. We infer a two-terminal field-effect hole mobility of 1600 cm^2^ V^−1^ s^−1^ and electron mobility of 220 cm^2^ V^−1^ s^−1^ at *V*_bg_ = 0 V. The mobility is estimated in the linear region of the transfer curve using *μ* = *L*/*W* ⋅ 1/(*C*_tg_ *V*_ds_) ⋅ d*I*_ds_/d*V*_tg_, where *L* = 1.6 μm and *W* = 25 μm is the length and the total width of the device, respectively, *I*_ds_ is the source-drain current and *C*_tg_ = *ε*_t_ *ε*_0_/*d*_t_ is the top gate capacitance per unit area. Here, *ε*_0_ is the permittivity of vacuum, and *ε*_t_ and *d*_t_ (23 nm) are the relative permittivity and thickness of the top hBN dielectric, respectively. In the dual-gate configuration, two displacement fields generated in top (*D*_t_ = *ε*_t_(*V*_tg_ − *V*_*t*0_)/*d*_t_) and back (*D*_b_ = *ε*_b_(*V*_bg_ − *V*_*b*0_)/*d*_b_) gate dielectrics are utilized to control the doping and potential difference across thin BP films, where *ε*_b_ is the overall relative permittivity of back gate dielectric (including 90 nm SiO_2_ and a 9 nm-thick bottom hBN flake), *d*_b_ (99 nm) is the total thickness of back gate dielectric, and *V*_t0_ and *V*_b0_ are charge-neutrality point voltages due to the unintentional doping. We find a small *V*_t0_ value (~0.5 V), which indicates that the BP sample is very intrinsic. Figure 2a shows source-drain current *I*_ds_ as function of top gate voltages with source-drain bias of 100 mV at different static back gate bias *V*_bg_. With decreasing back gate bias, the charge-neutrality point of BP shifts to higher top gate bias and scales linearly with *V*_bg_ (Supplementary Fig. 4), indicating that two gates with opposite signs are able to effectively compensate the doping. At charge-neutrality points, the displacement fields across BP channel satisfy the relation *D* = *D*_t_ = *D*_b_. From this relation, the relative permittivity of hBN is estimated to be ~3.1 in our devices. This value is consistent with the results reported previously^29^. As shown in Fig. 2a, the minimum conductance at charge-neutrality point increases significantly at higher displacement field, clearly indicating the bandgap decrease. A quantitative study of the bandgap reduction as function of the displacement field has previously been performed using temperature-dependent four-terminal conductance measurements at charge-neutrality points^23^ or using the scanning tunneling microscope^25^. In addition, the bandgap tuning in few-layer BP has also been explored theoretically and using angle-resolved photoemission spectroscopy^23, 25, 30–34^.

Here, in order to theoretically explore the impact of the vertical electric bias on the light-BP interaction, we include the crystal-momentum dependence in a tight-binding model^23^ to describe the single-particle optical conductivity, based on the Kubo formula in thin BP films. The detailed modeling process of the BP optical conductivity under bias is presented in Supplementary Note 2. In this model, the change of optical oscillator strength due to the bias-induced separation of electron and hole wave functions is taken into account, leading to optical conductivity within single-particle framework. Figure 2b shows the calculated results of the optical absorption edge, the energy at which the increase of the optical conductivity is the sharpest (Supplementary Fig. 5), as a function of displacement field in 9-, 10-, and 11-layer BP films.

### Tunable mid-IR photoresponse in the 10-layer BP device

**Fig. 3:**
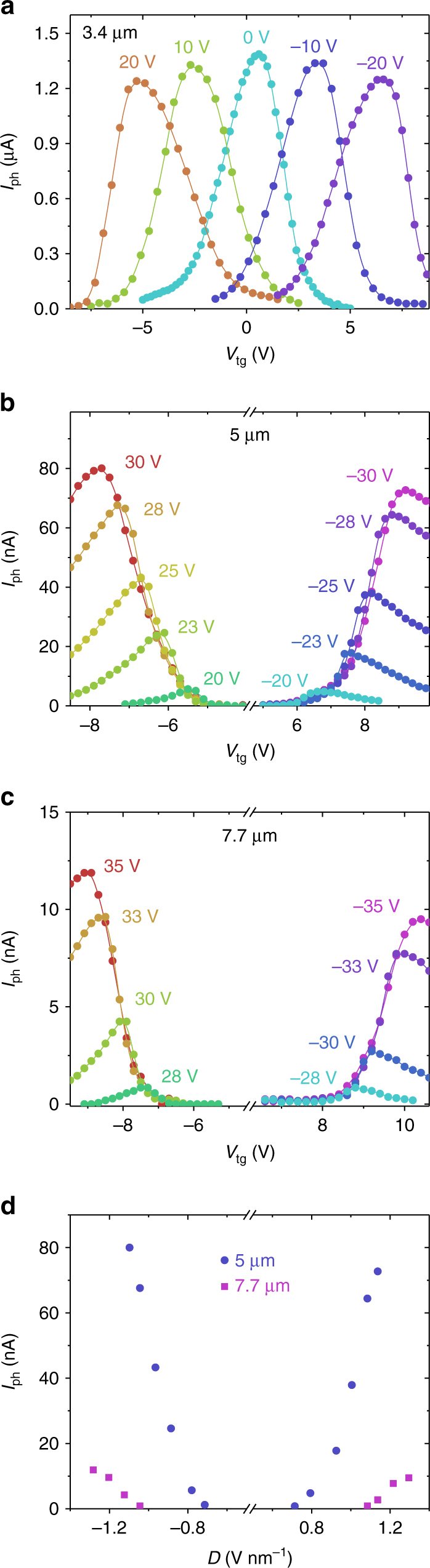
Tunable photoresponse in the dual-gate 10-layer mid-IR BP photodetector. **a**–**c** Photocurrents as function of top gate biases at different static back gate biases for 3.4 (**a**), 5 (**b**), and 7.7 μm (**c**) incident lasers. The incident laser powers are 40, 50, and 100 μW for 3.4, 5, and 7.7 μm wavelengths, respectively. The source-drain voltage is 300 mV. **d** Peak photocurrents at different displacements fields for 5 and 7.7 μm incident lasers

Next we measured the tunable photoresponse of the 10-layer BP photodetector under the vertical displacement field at three representative mid-IR wavelengths, 3.4, 5, and 7.7 μm at 77 K. The IR light was chopped and focused on samples to generate an alternating photocurrent (*I*_ph_), which was then collected by a lock-in amplifier referenced to the chopping frequency (details are further shown in Methods) at a bias voltage *V*_ds_ of 300 mV. At 3.4 μm (photon energy is above the bandgap of unbiased BP), the hBN-sandwiched BP device always shows maximum photocurrents at charge-neutrality points (Fig. 3a) regardless of the back gate bias *V*_bg_, which is distinctively different from previous studies^13, 19^ in which the photo-gating effect dominates the photocurrent due to the presence of significant trap states. With increasing carrier doping, the photocurrent decreases dramatically, primarily due to higher photo-carrier scattering rate and hence reduced carrier lifetime. This is the key signature of the photoconductive effect^14^, in which photo-generated electrons and holes drift in opposite directions and are eventually collected by drain and source leads. At *V*_ds_ = 300 mV, we achieve an extrinsic responsivity of 136 mA W^−1^ at the charge-neutrality point at *V*_bg_ = 0 V. The extrinsic responsivity is defined as *R*_ex_ = *I*_ph_/(*P*_in_ *A*_device_/*A*_laser_), where *P*_in_ is the incident laser power, *A*_device_ is the effective sample area, and *A*_laser_ is the laser spot area. At higher displacement fields, the peak photocurrent decreases slightly. This can be due to two mechanisms. First, under bias the absorption edge tuning leads to re-distribution of the oscillator strength. This can lead to the reduction of oscillator strength at the transition energy corresponding to 3.4 μm light (photon energy of 365 meV). Second, bandgap shrinkage results in the increase in the free intrinsic carrier density, which can reduce the photo-carrier lifetime, leading to the reduced photoresponse.

In Fig. 3b, we plot the top gate-dependent photocurrent at different static back gate bias under illumination of the 5 μm mid-IR light (with photon energy of 248 meV) with polarization along the *X*-direction of BP. We start to observe significant photocurrent when |*V*_bg_| > 20 V, corresponding to a displacement field |*D*| > 0.78 V nm^−1^ in BP when charge-neutrality condition is achieved. Again, at any given back gate bias beyond the threshold voltage of around 20 V, the photocurrent peaks when charge-neutrality condition is satisfied. Moreover, the peak photocurrent increases significantly with increasing displacement field and reaches ~80 nA at a displacement field of 1.14 V nm^−1^ (*V*_bg_ = −30 V, *V*_tg_ = −7.7 V). This yields an extrinsic responsivity of around 8 mA W^−1^ at *V*_ds_ = 300 mV. We further examine the photoresponse under the 7.7 μm light excitation in Fig. 3c. The observation of photocurrent clearly indicates efficient light-BP interactions at 7.7 μm (with photon energy of 160 meV) when |*D*| > 1.09 V nm^−1^. We further plot the peak photocurrent at the charge-neutrality condition as a function of the displacement field in BP in Fig. 3d. We obtain the threshold displacement fields (−0.73 and −1.07 V nm^−1^ at hole side, and 0.73 and 1.11 V nm^−1^ at electron side) at which the optical absorption edge (defined as the energy at which the optical conductivity increase is the largest) of the 10-layer BP shrinks to 250 and 160 meV, respectively. We plot these data in Fig. 2b and find excellent agreement between experimental results and theoretical predictions for the 10-layer BP. As discussed in Supplementary Note 2, at zero bias we assume that the absorption edge in BP thin film is its bandgap (330 meV). Here we want to mention that the theoretical results on the tuning of optical absorption edge are in fact similar to the values for bandgap edge tuning^23^. However, the theoretical results on optical conductivity shown in Supplementary Fig. 5 provide additional information on the strength of the light-matter interaction in BP under bias. As discussed below and in Supplementary Note 2, although in biased BP smaller photoresponse for 5.5 and 7 μm light is observed, if compared with 3.4 μm light, whose photon energy is above the unbiased BP bandgap of 330 meV, theoretical calculations indicate that the optical conductivity at low energy in biased BP can still be significant.

**Fig. 4:**
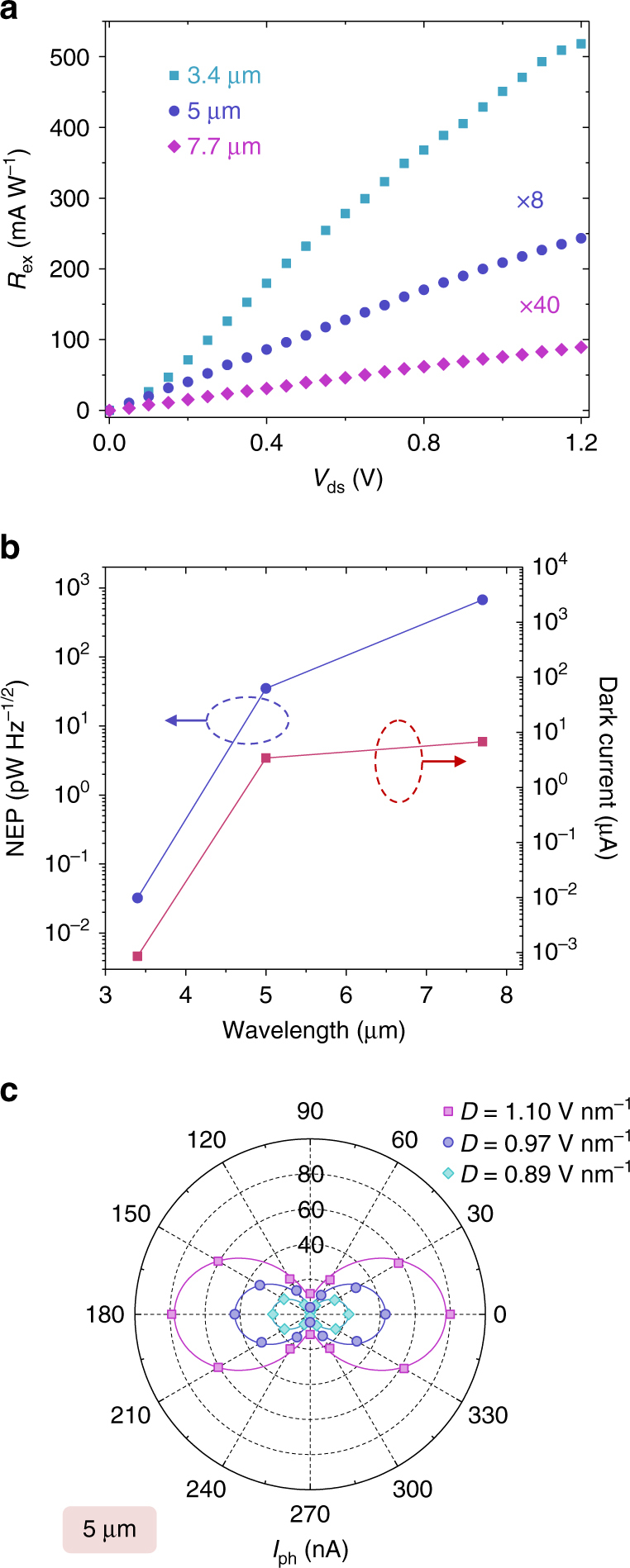
Photo-responsivity and polarization-sensitivity in 10-layer mid-IR BP photodetector. **a** Photo-responsivity as a function of source-drain bias at charge-neutrality points. For 3.4, 5, and 7.7 μm incident lasers, the back gate bias is 0, 30, and 35 V, respectively, and the incident laser powers are 40, 50, and 100 μW, respectively. **b** NEP and dark currents at charge-neutrality points at the three representative mid-IR wavelengths. For photocurrent measurements at 3.4, 5, and 7.7 μm, the back gate bias is 0, 30, and 35 V, respectively. **c** Scatters show the photocurrent at different laser polarizations for the 5 μm incident laser. The incident power is 50 μW. Here the incident angle of 0° corresponds to the laser polarization aligned with the *X*-direction (armchair) of BP. Solid lines are fitting curves from the equation $I = \left( {I_{{\mathrm{max}}} - I_{{\mathrm{min}}}} \right){\mathrm{cos}}{}^2\theta + I_{{\mathrm{min}}}$, where *θ* is the polarization angle referenced to the *X*-direction of BP, and *I*_max_ and *I*_min_ are the photocurrent intensities along *X*- and *Y*-direction, respectively

Figure 4a plots the extrinsic responsivity as a function of source-drain bias at 3.4, 5, and 7.7 μm wavelengths, respectively. The device is operated at the charge-neutrality point at back gate bias of 0, 30, and 35 V for the three wavelengths, respectively. The responsivity increases linearly with *V*_ds_ when *V*_ds_ < 1 V, and only shows very slight saturation at larger *V*_ds_. This can be attributed to a higher carrier drift velocity at larger *V*_ds_, which can be expressed as *μV*_ds_/*L*. At a source-drain bias of 1.2 V, the device exhibits an extrinsic responsivity of 518, 30, and 2.2 mA W^−1^ at 3.4, 5, and 7.7 μm, respectively. If taking into account the optical absorption of Pt top gate film (40%) and BP (~3% for 5 nm-thick thin film)^35^, the intrinsic responsivity (*R*_in_) are estimated to be 28.7, 1.6, and 0.122 A W^−1^ at 3.4, 5, and 7.7 μm, respectively. As shown in the Supplementary Fig. 5, the optical conductivity of biased BP at low photon energy is in general smaller than that at high photon energy (above 330 meV). The light absorption of 5 and 7.7 μm photons in 5 nm-thick BP under bias is below 3%. As a result, the estimated intrinsic responsivity at 5 and 7.7 μm are lower bound values due to the smaller absorption coefficients at longer wavelength. At 3.4 μm, the product of internal quantum efficiency *IQE* and photoelectric current gain *G*, defined as *IQE* ⋅ *G* = *R*_in_ *E*_ph_/*e*, reaches 10.5 at *V*_ds_ = 1.2 V, where *E*_ph_ is the photon energy and *e* is the elementary charge. This high *IQE* ⋅ *G* is attributed to a short carrier transit time *τ*_tr-h_ = *L* ^2^/*μ*_h_ *V*_ds_ ~ 13 ps for holes and *τ*_tr-e_ ~ 97 ps for electrons in our hBN-sandwiched BP devices. Assuming a unity electron-hole pair generation possibility, we can estimate the photo-carrier lifetime^36^ *τ*_life_ = *τ*_tr-h_ *τ*_tr-e_/(*τ*_tr-h_ + *τ*_tr-e_) ⋅ *IQE* ⋅ *G* to be around 120 ps at the charge-neutrality point. This value agrees very well with previously reported value of ~100 ps using time-resolved reflection measurements^37^. Since the photo-carrier lifetime is well below nanosecond, the optoelectronic devices based on tunable BP can potentially operate at a speed beyond 1 GHz. The estimated 3-dB bandwidth *f*_3dB_ of the operation in our current device at charge-neutrality point is around 1.3 GHz (*f*_3dB_ = 1/2*πτ*_life_)^38^, if the RC time constant of the device is much shorter than the carrier lifetime. Although tuning the device away from the charge-neutrality point leads to reduced photo-carrier lifetime and hence lower quantum efficiency as shown in Fig. 3a–c, the reduced photo-carrier lifetime can result in an increase in operational bandwidth, which is desirable for high speed applications.

Another important figure of merit for detectors is the noise equivalent power (NEP)^36, 39^. It denotes the minimum required incident light power to achieve power signal to noise ratio of 1 $( {I_{{\mathrm{ph}}}^2{\mathrm{/}}\langle {i_{\rm n}^2} \rangle = 1} )$ at 1 Hz bandwidth^36^. Here $\sqrt {\langle {i_{\rm n}^2} \rangle }$ is the noise current and $\langle {i_{\rm n}^2} \rangle$ can be attributed to the sum of the shot noise ($\langle {i_{\rm s}^2} \rangle = 2eI_{{\mathrm{dark}}}$ at 1 Hz bandwidth), Johnson noise ($\langle {i_{\rm j}^2} \rangle = 4k_{\mathrm{B}}TI_{{\mathrm{dark}}}{\mathrm{/}}V_{{\mathrm{ds}}}$ at 1 Hz bandwidth, where *k*_B_ is the Boltzmann constant and *T* = 77 K is the device operating temperature), and generation-recombination noise currents ($\langle {i_{{\rm gr}}^2} \rangle = 4eI_{{\mathrm{ph}}}( {\tau _{{\mathrm{life}}}{\mathrm{/}}\tau _{{\mathrm{tr}}}})$ at 1 Hz bandwidth when the operating frequency $f \ll 1{\mathrm{/}}\tau _{{\mathrm{life}}}$) in photoconductors^36^. As the operating frequency in our tunable BP device is well beyond 10 kHz as shown in Supplementary Fig. 6, we neglect the contribution from 1/*f* flicker noise, which is only significant at low operating frequencies (<10 kHz)^19, 40^. By taking into account these three noise mechanisms, the NEP at the three mid-IR wavelengths is extracted and plotted in Fig. 4b. Here the device is operated at the charge-neutrality point at back gate bias of 0, 30, and 35 V for 3.4, 5, and 7.7 μm wavelengths, respectively, where the NEP is optimal. Detailed calculation of NEP is shown in Supplementary Note 3. The device shows a NEP of 0.03, 35, and 672 pW Hz^−1/2^ at 3.4, 5, and 7.7 μm wavelengths, respectively, at *V*_ds_ = 1.2 V. The dark currents are 8.6 × 10^−4^, 3.42, and 6.75 μA for the three wavelengths, respectively, as shown in Fig. 4b. The NEP of our tunable BP photodetector at 3.4 μm is better than that obtained in b-AsP FETs (~2 pW Hz^−1/2^) and b-AsP/MoS_2_ heterojunctions (~0.2 pW Hz^−1/2^)^22^. For longer wavelengths, the NEP increases to 35 pW Hz^−1/2^ at 5 μm and 672 pW Hz^−1/2^ at 7.7 μm, due to smaller responsivity and higher operating darker current. These values are larger than that reported in b-AsP-based devices^22^. However, our tunable BP photodetectors show advantages of potential operating frequency of gigahertz, which is significantly larger than that (~kHz) in b-AsP devices^22^ and our devices have unique spectral tunability. On the other hand, the b-AsP devices have the advantage of room-temperature operation.

If compared with the state-of-the-art wide-band mid-IR detectors, such as mercury cadmium telluride (HgCdTe)-based detectors with NEP below 10^−6^ pW Hz^−1/2^ for ~10 μm IR light operating at 260 K^41^, the NEP of our tunable BP device is much larger. However, our demonstration is based on an ultrathin BP film (~5 nm) in which the light absorption is around 3% for 3.4 μm light and even smaller for 5 and 7.7 μm light. Further integration with optical structures (such as cavities and waveguides) is expected to significantly improve its performance. Moreover, our photodetectors have widely tunable spectral response and can be integrated with electronics readily due to the layered and nontoxic nature of BP. In short, although the device performance reported here is still not as good as state-of-the-art HgCdTe photodetectors, there is still plenty of room for the improvement. More importantly, our devices have many unique features such as wide tunability and potential for monolithic integration, which are not available in traditional HgCdTe photodetectors.

The photoresponse of BP thin films also shows strong angle-dependence at all three representative mid-IR wavelengths. Figure 4c shows the photoresponse at 5 μm with light polarization at different angles as an example. The responsivity reaches the maximum value when light polarization is along the *X*-direction (armchair), while reaches the minimum value along the *Y*-direction. This is attributed to the small optical absorption coefficient along the *Y*-direction of BP originating from its unique puckered lattice structure^42^.

## Discussion

The ability to tune the photoresponse of BP in a wide mid-IR spectral range implies that BP could be an alternative material for mid-IR photonics. The demonstration of tuning here is also distinctively different from previously results on BP optical property tuning with a single gate, in which both the Franz–Keldysh and Pauli-blocked Burstein–Moss effects^43–45^ play a role. The Burstein–Moss effect^43, 44^ in highly doped BP leads to a blueshift of optical absorptions at high charge carrier densities due to blockings of low-energy optical transitions by band filling. The Franz–Keldysh effect^43–45^ redshifts the BP absorption edge when the doping of BP is below ~3 × 10^12^ cm^−2^, while the Burstein–Moss dominates and blueshift of the absorption is expected at higher doping concentrations^43, 44^. Here the dual-gate configuration provides an additional gate variable and allows us to reach the charge-neutrality position of BP at different biasing field, leading to significant redshift of the light absorption edge, highly desirable for mid-IR applications. Moreover, in our 10-layer BP, the exciton binding energy is negligibly small and excitonic effect can be ignored^3^.

In summary, a widely tunable mid-IR photodetector based on the hBN/BP/hBN heterostructure has been demonstrated for optoelectronic applications beyond the cutoff wavelength of pristine BP. Strong photoresponse generated from intrinsic photoconductive effect is observed at wavelengths up to 7.7 μm under a moderate displacement field. High intrinsic mobility and strong photoresponse in a broad mid-IR wavelength range, together with its layered nature make BP a promising material for high-speed mid-IR photodetectors, modulators, and waveguide-integrated on-chip spectrometers.

## Methods

### Fabrication of hBN-sandwiched BP mid-IR photodetectors

BP crystals are purchased from HQ Graphene with purity >99.995%, synthesized by the Sn/SnI_4_ approach^46, 47^. BP and hBN thin flakes were first mechanically exfoliated from bulk crystals onto silicon substrates covered with a 90 nm-thick SiO_2_ in an argon-filled glovebox with oxygen and moisture concentrations lower than 0.1 ppm. The hBN/BP/hBN heterostructures were assembled using the polymer-free dry transfer method described in ref. ^27^. The heterostructure stack was annealed at 633 K in a hydrogen/argon (flow ratio 3:97) environment at atmosphere pressure (1 atm) for 6 h to further improve its quality. Before the fabrication of electrodes, a polarization-resolved Horiba HR LabRaman 300 system (532 nm laser as the excitation) was used to characterize the crystal direction of BP. Then a 200 nm PMMA was spun onto samples and a Vistec 100 kV electron-beam lithography system was used to define the shape of multiple interdigitated electrodes. The exposed top hBN layer was etched using the Oxford Plasmalab 100 Reactive Ion Etching System in a CHF_3_/O_2_ (40/4 standard cubic centimeter per minute) environment. Chromium/gold (3/27 nm) films were then evaporated to form contacts. Another BN flake was transferred to cover the whole sample area. At last a 7 nm-thick Pt top gate was evaporated after electron-beam lithography to fully cover the BP channel.

### Transmission electron microscopy characterizations

Following thermal evaporation of 6–10 nm of amorphous carbon on the sample for improved conductivity, a thin device cross-section lamella was prepared using a focused Ga-ion beam in a FEI Helios DualBeam machine. HR-TEM imaging was performed using an FEI Tecnai F-30 instrument. The lamella was exposed to air for < 5 min during transfer to the HR-TEM. Images were acquired at a working voltage of 300 kV. Inter-layer distances of different strata in the hBN/BP/hBN stack were obtained from fast Fourier transform of selected areas in the device cross-section. Elemental mapping of the device cross-section was performed using electron energy loss spectroscopy in combination with a Gatan imaging filter, yielding an energy-filtered transmission electron microscopy image.

### Transport and mid-IR photocurrent measurements

The dual-gate transport measurements were performed using an Agilent B1500A semiconductor parameter analyzer in a low-temperature stage (model HFS600E-PB4 from Linkam Scientific Instruments) mounted on a Bruker Fourier Transform Infrared spectrometer (FTIR). Chopped light from a helium-neon laser (3.4 μm) and quantum cascade lasers (5 and 7.7 μm) was coupled to the FTIR and then focused on the samples using the Hyperion 2000 microscope. The generated alternating photocurrent was then collected by a Lock-in amplifier (Model SR830) referenced to the chopping frequency of 1.3 kHz. The diameters of the laser spot for 3.4, 5, and 7.7 μm lasers are ~14, 16, and 16 μm, respectively.

### Data availability

The data that support the findings of this study are available from the corresponding author upon reasonable request.

## Electronic supplementary material

Supplementary Information
