# Supplementary material for: Widely tunable black phosphorus mid-infrared photodetector
Source: Nat Commun. 2017 Nov 22;8:1672. doi: 10.1038/s41467-017-01978-3 (PMC5698317; doi:10.1038/s41467-017-01978-3)
Supplement: Supplementary file 1 — Supplementary Information [file 41467_2017_1978_MOESM1_ESM.pdf]

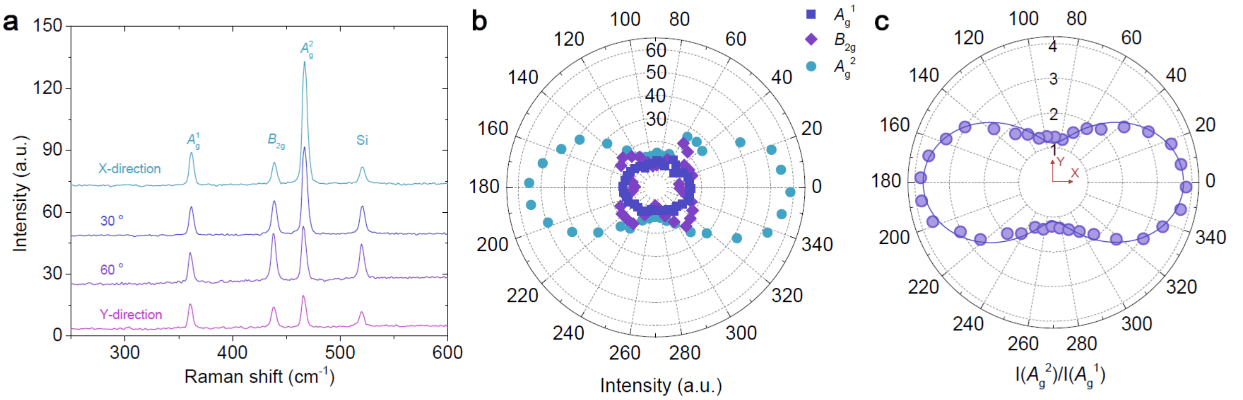

Supplementary Figure 1 | Raman characterization of BP thin films. **a**, Raman spectra of the 5 nm-thick BP sandwiched by hexagonal boron nitride (hBN) with excitation laser (532 nm) polarizations aligned at different incident angles. **b**, The intensities of A_g^1, B_{2g} and A_g^2 peaks as function of laser polarization angles. **c**, The ratio of A_g^2 and A_g^1 peak intensity (scatters) fitted by the relation $I=(I_{max}-I_{min})\cos^2\theta+I_{min}$ (solid line).

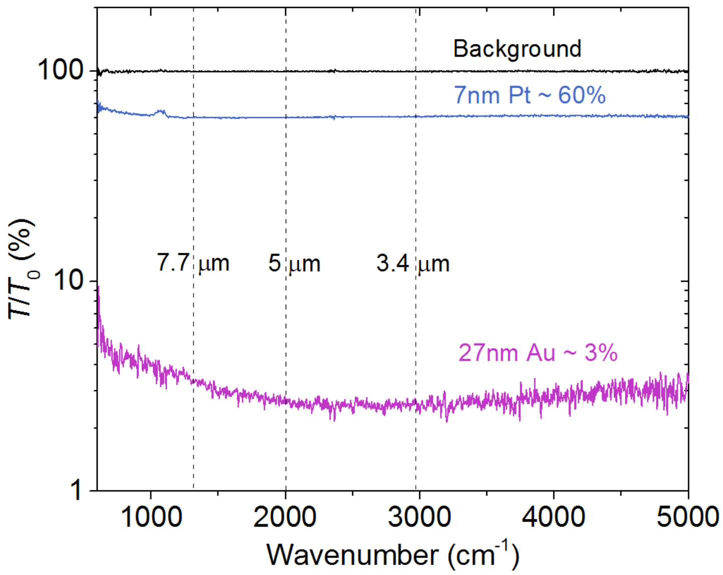

Supplementary Figure 2 | Transmission curves of the 7 nm-thick platinum (Pt) and 27 nm-thick gold (Au) thin films. The transmission efficiency is around 60% at 3.4, 5 and 7.7μm for the 7 nm-thick Pt film. Only 3% mid-infrared light transmits through the 27 nm-thick gold film. As a result, the infrared light absorption in BP underneath the source and drain electrodes can be ignored.

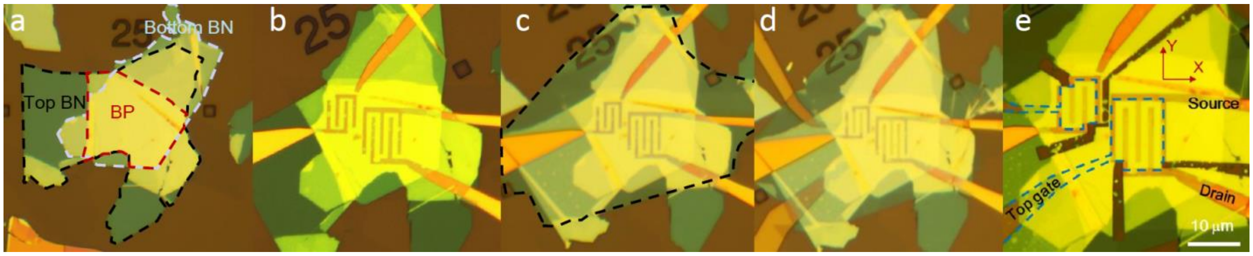

Supplementary Figure 3 | Dual-gate black phosphorus (BP) transistor fabrication process. **a.** The hBN/BP/hBN heterostructure is assembled using the polymer-free dry transfer method [1]. Annealing at 633 K for 6 hours further stabilizes the heterostructure. **b.** Electron-beam lithography is used to define the Poly-methylmethacrylate (PMMA) mask for etching and electrode contacts. The exposed top BN layer is etched away in a reactive ion etching system. Then chromium/gold (3/27 nm) electrode is evaporated on samples to form contacts. **c.** Another hBN layer is transferred onto samples as indicated by the dashed line enclosed area. **d.** The second electron-beam lithography is performed to define the shape of top gate. A 7-nm thick Pt film is then deposited. **e.** The final electron-beam lithography and etch step form trenches to electronically isolate different devices.

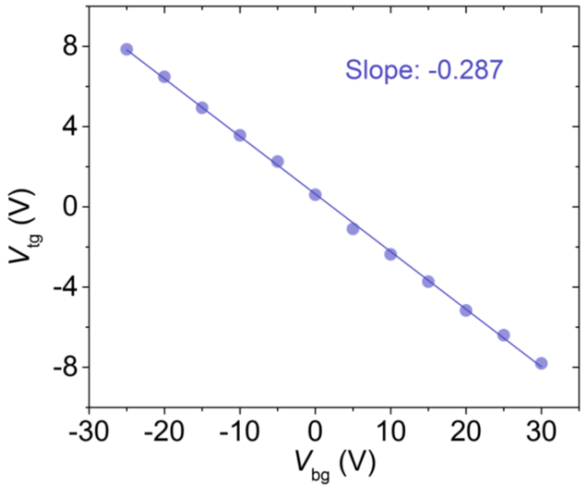

Supplementary Figure 4 | Linear relationship of V_{tg} and V_{bg}. The scatters show the top gate bias at charge neutrality points as a function of back gate bias. The solid line is the linear fitting that shows a slope of -0.287, from which the dielectric constant of hBN is inferred.

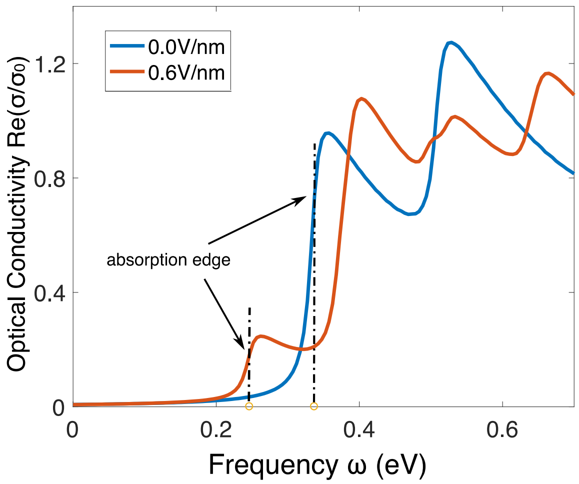

Supplementary Figure 5 | Theoretical calculation of the optical conductivity. The optical conductivities under 0.0 V nm^{-1} (blue) and 0.6 V nm^{-1} (red) displacement fields in armchair direction. The absorption edges are indicated by dashed lines. σ_0 denotes a universal conductance of $e^2/4\hbar$, where e is the elementary charge and $\hbar$ is the reduced Planck's constant.

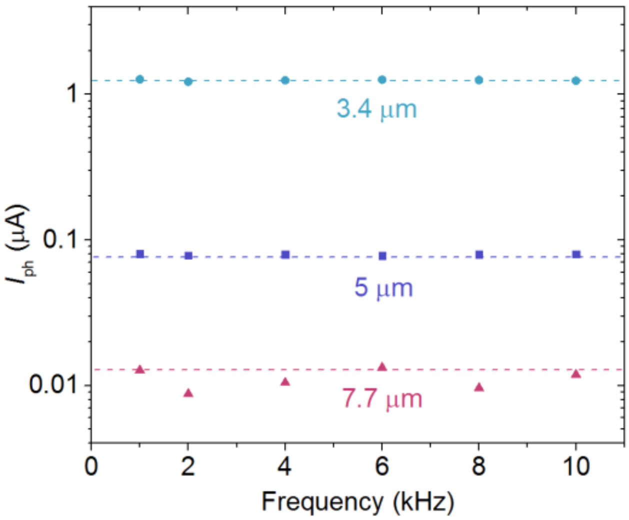

Supplementary Figure 6 | Frequency-dependence of the photocurrent. Measured photocurrents at the three mid-infrared wavelengths do not show trend of decline up to chopping frequency of 10 kHz. The device is characterized at charge-neutrality points with back gate biases of 20, 30 and 35 V, respectively.

Supplementary Note 1. Raman spectra of thin BP films sandwiched by hBN.

Fig. S1a shows the Raman spectra of the 10-layer BP film sandwiched by hBN with linearly polarized excitation laser (532 nm) whose polarization is aligned at different incident angles. The 0° incident angle indicates that the polarization of the excitation laser is along the X-direction (armchair) of BP. The spectra show three typical phonon modes of BP at around 361, 439 and 467 cm^{-1} (A_g^1, B_{2g} and A_g^2 peaks), respectively [3]. The intensities of A_g^1, B_{2g} and A_g^2 peaks at different polarization angles are shown in Supplementary Fig. 1b. The A_g^2 peak intensity strongly depends on the laser polarization in contrast to that of A_g^1 peak. This is because that A_g^2 peak is related to armchair-oriented phonon vibration mode, while the A_g^1 peak corresponds to the phonon vibration out-of-plane [3]. The A_g^2 peak intensity reaches its maximum value when excitation laser polarization is along the X-direction of BP. B_{2g} peak shows 4 maximum intensities at D-direction (45° respect to the X-direction) and reaches minimum values at X and Y-directions [3]. The observations are consistent with previous Raman studies on thin BP films (with thickness smaller than 15 nm) using the 532 nm laser [3]. We notice that the peak intensity values near 50° and 60° incident angles in Fig. S1b deviate slightly from the trends discussed above, which is probably due to the minor instability of the laser intensity. However, we find that the ratio of A_g^2 and A_g^1 peak intensity ($I(A_g^2)/I(A_g^1)$) can eliminate the effect of laser power instability and results in a nice stable trend, as demonstrated in Supplementary Fig. 1c here and Fig. 1d in the main text. The curve can be well fitted by the relation $I=(I_{\max}-I_{\min})\cos^{2}\theta+I_{\min}$, where θ is the laser polarization angle referenced to the X-direction of BP, and I_{max} and I_{min} denotes the maximum and minimum value of $I(A_g^2)/I(A_g^1)$.

Supplementary Note 2. Theoretical calculation of the optical conductivity of biased BP thin films

To capture the optical response of BP thin films around the Γ point, we have developed a tight-binding model based on density functional theory (DFT) in conjunction with the $k\cdot p$ approximation. The effective Hamiltonian of monolayer BP can be described by a simple two-band model [4,5]:

$$H=\begin{pmatrix} E_c+\alpha k_x^2+\beta k_y^2 & v_f k_y \\ v_f k_y & E_v-\lambda k_x^2-\mu k_y^2 \end{pmatrix} \quad (1)$$

where E_c and E_v are energies of the conduction and valence band edge at the Γ point, respectively. k_x and k_y are components of the crystal momentum along armchair (x) and zigzag (y) directions, respectively. $\alpha,\beta,\lambda,\theta$ and μ are parameters related to effective masses in different crystalline directions and v_f is the Fermi velocity of the nearly linear band along the armchair direction. Here, $\alpha=0.027$ eVnm^2, $\beta=0.253$ eVnm^2 $\lambda=0.033$ eVnm^2, $\mu=0.313$ eVnm^2, $\theta=0.026$ eVnm^2 and $v_f=0.5344$ eVnm are parameters which are fitted by six layer BP DFT results.

To calculate the optical conductivity of multilayer BP under electric fields, we further introduce the interlayer coupling coefficients and electrostatic potential of each layer under the tight-binding framework [5]. Then the low-energy Hamiltonian of N-layer BP can be written as:

$$\begin{pmatrix} E_c(k) & t_1 & t_2 & \cdots & t_n & v_f k_y & 0 & 0 & \cdots & 0 \\ t_1 & E_c(k)+\Delta & t_1 & & t_{n-1} & 0 & v_f k_y & 0 & & 0 \\ t_2 & t_1 & E_c(k)+2\Delta & & t_{n-2} & 0 & 0 & v_f k_y & & 0 \\ \vdots & & & \ddots & \vdots & \vdots & & & \ddots & \vdots \\ t_n & t_{n-1} & t_{n-2} & \cdots & E_c(k)+(n-1)\Delta & 0 & 0 & 0 & \cdots & v_f k_y \\ v_f k_y & 0 & 0 & \cdots & 0 & E_v(k) & t_1' & 0 & \cdots & 0 \\ 0 & v_f k_y & 0 & & 0 & t_1' & E_v(k)+\Delta & t_1' & & 0 \\ 0 & 0 & v_f k_y & & 0 & 0 & t_1' & E_v(k)+2\Delta & & 0 \\ \vdots & & & \ddots & \vdots & \vdots & & & \ddots & \vdots \\ 0 & 0 & 0 & \cdots & v_f k_y & 0 & 0 & 0 & \cdots & E_v(k)+(n-1)\Delta \end{pmatrix} \quad (2)$$

where $E_c(k) = E_c + \alpha k_x^2 + \beta k_y^2$ and $E_v(k) = E_v - \lambda k_x^2 - \mu k_y^2$. As discussed in Ref. [5], t_i $(i = 1, \ldots, n)$ represents the interlayer coupling strength between conduction bands. t_1 is the nearest neighboring interlayer coupling strength, and t_i is the ith nearest neighboring interlayer coupling strength. Here we set $t_1 = 142$ meV and $t_2 = -85$ meV based on Ref. [5]. The terms beyond t_2 are small enough to be ignored. For valence bands, we only need to consider the nearest coupling strength t'_1(-318 meV). Here we use the negative value for t'_1 due to the phase difference between conduction and valence electron wave functions.

Our model captures the effect of an external electric field by introducing an extra electrostatic potential difference Δ of each layer. Δ is defined as $\Delta = \frac{E^{ext}}{\epsilon^*} \times d$, where E^{ext} is the external out of plane electric field in vacuum and d is the interlayer distance 0.55 nm. ϵ^* is the effective dielectric constant of BP film including the screening effect from the bandgap reduction, which is $\epsilon^* = \frac{E_0}{E_{\text{gap}}} \epsilon$ [6].

Then we apply the model to the Kubo formula to obtain the optical conductivity [5]:

$$\sigma_{ab}(q,\omega) = -i\frac{g_s \hbar e^2}{(2\pi)^2} \sum_{jj'} \int d\vec{k} \frac{f(E_{jk}) - f(E_{j'k'})}{E_{jk} - E_{j'k'}} \times \frac{< \Psi_{jk}|v_a|\Psi_{j'k'} >< \Psi_{j'k'}|v_b|\Psi_{jk} >}{E_{jk} - E_{j'k'} + \hbar\omega + i\eta} \quad (3)$$

where $\vec{v}_{a,b}$ is the velocity operator defined as $\hbar^{-1}\partial_{k_{a,b}}\mathcal{H}$, $g_s = 2$ is the spin degeneracy, and $\eta \approx 10\ meV$ accounts for the finite damping [4]. $E_{(j,j'),(k,k')}$ and $\Psi_{(j,j'),(k,k')}$ are eigenenergies and eigenfunctions solved from our low-energy effective Hamiltonian, respectively. We also set the temperature for Fermi-Dirac distribution to be 77 K, at which our experiments are performed. Here we only consider the vertical interband optical transition between conduction and valence bands.

Using this method, we have calculated the optical conductivity of the 9-, 10- and 11-layer BP films under different displacement fields. In Supplementary Fig. 5, the real part of the optical conductivity along the armchair direction of a 10-layer BP film is plotted under two different gating fields. As shown in Supplementary Fig. 5, the optical absorption edge is tuned significantly by the field. We define the optical absorption edge as the energy at which the single-particle optical conductivity (real part) has the sharpest slope. At zero bias, the absorption edge of 10-layer black phosphorus is simply its bandgap (330 meV). The evolution of the optical

absorption edge according to the biasing field is presented in Figure 2b of the main text, which shows an excellent agreement with experiments.

Finally, we want to emphasize that this calculation is based on the single-particle transport picture, which neglects complicated scattering mechanisms. As a result, although the optical absorption edge is reliable, the higher-energy features and the magnitude of the optical conductivity in Supplementary Fig. 5 can be affected by the scattering and recombination of electrons and holes, which are also field-dependent.

Supplementary Note 3. Calculation of noise equivalent power (NEP)

As discussed in Ref. [2], the NEP is the incident power to achieve a power signal to noise ratio of 1 ($I_{\rm ph}^2 / < i_n^2 >= 1$) at 1Hz bandwidth, where $< i_{\rm n}^2 >$ is the sum of the shot noise ($< i_{\rm s}^2 >= 2eI_{\rm dark}$ at 1 Hz bandwidth, where e is the elementary charge and $I_{\rm dark}$ is the dark current), Johnson noise ($< i_{\rm j}^2 >= 4k_{\rm B}TI_{\rm dark} / V_{\rm ds}$ at 1 Hz bandwidth, where $k_{\rm B}$ is the Boltzmann constant, T is the temperature and $V_{\rm ds}$ is the source-drain voltage) and generation-recombination noise currents ($< i_{\rm gr}^2 >= 4eI_{\rm ph}(\tau_{\rm life} / \tau_{\rm tr})$ at 1 Hz bandwidth, where $I_{\rm ph}$ is the photocurrent and $\tau_{\rm life}$ and $\tau_{\rm tr}$ denote the life and transit time of photocarriers, respectively). By substituting $< i_{\rm n}^2 >=< i_{\rm s}^2 > + < i_{\rm j}^2 > + < i_{\rm gr}^2 >$ and $I_{\rm ph} = R_{ex} \cdot NEP$ into the equation $I_{\rm ph}^2 / < i_n^2 >= 1$, we have $NEP^2 - \frac{4e\tau_{\rm life}}{R_{\rm ex}\tau_{\rm tr}} NEP = \frac{2eI_{\rm dark}}{R_{\rm ex}^2} + \frac{4k_{\rm B}TI_{\rm dark}}{V_{\rm ds}R_{\rm ex}^2}$. Here, $R_{\rm ex}$ denotes the extrinsic responsivity. By solving this equation, we get $NEP = \frac{1}{R_{\rm ex}}(\frac{2e\tau_{\rm life}}{\tau_{\rm tr}} + \sqrt{2eI_{\rm dark} + \frac{4k_{\rm B}TI_{\rm dark}}{V_{\rm ds}} + \frac{4e^2\tau_{\rm life}^2}{\tau_{\rm tr}^2}})$. Because $\frac{4e^2\tau_{\rm life}^2}{\tau_{\rm tr}^2} << 2eI_{\rm dark}$ and $\frac{4k_{\rm B}TI_{\rm dark}}{V_{\rm ds}}$, the noise currents contributed by generation-recombination noise is far smaller than shot noise and thermal (Johnson) noise currents in our BP photodetectors based on the FET configuration. Hence, the NEP can be simplified as $NEP = \frac{1}{R_{\rm ex}} \sqrt{2eI_{\rm dark} + \frac{4k_{\rm B}TI_{\rm dark}}{V_{\rm ds}}}$.

Supplementary References

1. Wang, L. *et al*. One-dimensional electrical contact to a two-dimensional material. *Science* **342**, 614-617 (2013).

2. Chuang, S. L. Physics of photonic devices 2nd edn. (Wiley, 2009).

3. Kim, J. *et al.* Anomalous polarization dependence of Raman scattering and crystallographic orientation of black phosphorus. *Nanoscale* 7, 18708-18715 (2015).

4. Fei, R., Tran V. & Yang, L. Topologically protected Dirac cones in compressed bulk black phosphorus. *Phys. Rev. B* **91**, 195319 (2015).

5. Deng, B. *et al.* Efficient electrical control of thin-film black phosphorus bandgap. *Nature Commun.* **8**, 14474 (2017).

6. Low, T. *et al*. Tunable optical properties of multilayer black phosphorus thin films. *Phys. Rev. B* **90**, 075434 (2014).
